# Biochemical studies on a versatile esterase that is most catalytically active with polyaromatic esters

**DOI:** 10.1111/1751-7915.12107

**Published:** 2014-01-13

**Authors:** Mónica Martínez-Martínez, Iván Lores, Carlina Peña-García, Rafael Bargiela, Dolores Reyes-Duarte, María-Eugenia Guazzaroni, Ana Isabel Peláez, Jesús Sánchez, Manuel Ferrer

**Affiliations:** 1 Department of Applied Biocatalysis, Consejo Superior de Investigaciones Científicas (CSIC), Institute of CatalysisMarie Curie 2, 28049, Madrid, Spain; 2 Área de Microbiología, Departamento de Biología Funcional – IUBA, Universidad de OviedoJulián Clavería s/n, 33006, Oviedo, Spain; 3 Posgrado en Ciencias Naturales e Ingeniería, Universidad Autónoma MetropolitanaUnidad Cuajimalpa, Av. Vasco de Quiroga 4871, Col. Santa Fe, Deleg, Cuajimalpa, 05348, D.F, México; 4 Departamento de Procesos y Tecnología, Universidad Autónoma MetropolitanaUnidad Cuajimalpa, Av. Vasco de Quiroga 4871, Col. Santa Fe, Deleg, Cuajimalpa, 05348, D.F, México; 5 >Universidade de São Paulo, Faculdade de Filosofia Ciências e Letras de Ribeirão Preto, Departamento de QuímicaAvenida Bandeirantes 3900 Monte Alegre 14049-901, Ribeirao Preto, SP, Brasil

## Abstract

Herein, we applied a community genomic approach using a naphthalene-enriched community (CN1) to isolate a versatile esterase (CN1E1) from the α/β-hydrolase family. The protein shares low-to-medium identity (≤ 57%) with known esterase/lipase-like proteins. The enzyme is most active at 25–30°C and pH 8.5; it retains approximately 55% of its activity at 4°C and less than 8% at ≥ 55°C, which indicates that it is a cold-adapted enzyme. CN1E1 has a distinct substrate preference compared with other α/β-hydrolases because it is catalytically most active for hydrolysing polyaromatic hydrocarbon (phenanthrene, anthracene, naphthalene, benzoyl, protocatechuate and phthalate) esters (7200–21 000 units g^−1^ protein at 40°C and pH 8.0). The enzyme also accepts 44 structurally different common esters with different levels of enantio-selectivity (1.0–55 000 units g^−1^ protein), including (±)-menthyl-acetate, (±)-neomenthyl acetate, (±)-pantolactone, (±)-methyl-mandelate, (±)-methyl-lactate and (±)-glycidyl 4-nitrobenzoate (in that order). The results provide the first biochemical evidence suggesting that such broad-spectrum esterases may be an ecological advantage for bacteria that mineralize recalcitrant pollutants (including oil refinery products, plasticizers and pesticides) as carbon sources under pollution pressure. They also offer a new tool for the stereo-assembly (i.e. through ester bonds) of multi-aromatic molecules with benzene rings that are useful for biology, chemistry and materials sciences for cases in which enzyme methods are not yet available.

Microorganisms play a crucial role in soil genesis by facilitating mineralization not only for soil organic matter (Leigh Mascarelli, 2009) but also for prevalent and persistent pollutants, such as polyaromatic hydrocarbons (PAH) and heterocyclic aromatic compounds (Lu *et al*., 2011). Such compounds are common additives in crude oil and industrial chemical products, such as dyes, flavouring compounds, plasticizers, perfumes, pesticides and insect repellent as well as, more recently, microelectronics, printed circuit boards, silk screen printing devices, optical disks and black colour tube matrices (Kästner, 2000; Chae *et al*., 2002). Multiple microorganisms can obtain energy from such hydrophobic aromatics (Lu *et al*., 2011), which are primarily released into the environment through anthropogenic activities. When bacteria are confronted with aromatic compounds, the cells encounter an interesting contradiction. On the one hand, such chemical species can be mineralized to yield carbon and energy for growth, which allows microorganisms to colonize niches that are refractory to other microbes (Dominguez-Cuevas *et al*., 2006; Lu *et al*., 2011). On the other hand, aromatic compounds over a certain threshold are toxic for bacteria because they partition and disorganize the cell membrane by removing lipids and proteins, which leads to cell death (Sikkema *et al*., 1995; von Wallbrunn *et al*., 2003). To cope with such activities, bacteria have developed multiple chemical tolerance mechanisms and an extensive enzyme arsenal (e.g. oxygenases, *O*-demetylases, CoA synthases and ligases, aldolases, alcohol dehydrogenases and α/β hydrolases) for mineralizing aromatic compounds by submitting them to the central metabolism (Pérez-Pantoja *et al*., 2008; 2009; Vilchez-Vargas *et al*., 2013).

Recent studies suggest that multifunctional esterase/lipase-like proteins from the α/β hydrolase family that can hydrolyse both C-C and C-O bonds may exist in nature at much higher levels than previously thought (Alcaide *et al*., 2013). From an ecological perspective, such proteins may contribute to global carbon cycling processes for complex substrates, including recalcitrant organic pollutants. From a biotechnological perspective, such proteins may open unexpected research avenues for biotechnology applications. Esterases/lipases from the α/β hydrolase family have demonstrated such activities for catechol and biphenyl derivatives (Alcaide *et al*., 2013). However, the potential implication for the degradation of and biotechnology of complex organic molecules with two or more aromatic rings, specifically benzene rings (polyaromatic hydrocarbons – PAH), has not been described, even though the PAH degradation phenomenon is relatively well known (Pérez-Pantoja *et al*., 2008; 2009; Seo *et al*., 2009). Such activities are especially significant because the chemistry for PAH esters has been a subject of considerable interest due to their chemical and physical properties (e.g. polymers produced with versatile photo-reactivities) (Noh *et al*., 2001; Chae *et al*., 2002; Zhu *et al*., 2011). Heterogeneous catalysts have successfully been used to produce PAH-like (naphthalene, anthracene, phenanthrene, benzyl and phthalate) derivatives (Noh *et al*., 2001; Zhu *et al*., 2011; Maruyama *et al*., 2012). However, to the best of our knowledge, no study in the specialized literature has involved identification of potential applications for esterase/lipase-like proteins as catalysts for modifying PAHs.

Because contaminated environments and microbial communities derived therefrom are an excellent source for enzymes that act on aromatics (Pérez-Pantoja *et al*., 2008; 2009; Guazzaroni *et al*., 2013), we now ask whether such α/β hydrolases can be identified therein. We generated a subset of 5500 clones (in *Escherichia coli* EPI300-T1^R^; Epicentre Biotechnologies; Madison, WI, USA; Alcaide *et al*., 2013) from a naphthalene-enriched community (CN1) derived from PAH-contaminated soil (Guazzaroni *et al*., 2013), which included nearly 0.17 Gbp of community genomes, that were evaluated for their ability to hydrolyse α-naphthyl acetate (Reyes-Duarte *et al*., 2012). Four unique positive clones (hit rate 1:1375) were identified as active, and one (herein, CN1E1) was selected for in-depth analysis due to its high activity (halo/colour formation) and capacity for hydrolysis in agar-based assays using the model PAH ester naphthalene carboxylic acid methyl ester (Fig. 1). The insert [30 164 bp; nucleotide sequence available at the National Center for Biotechnology Information (NCBI) under accession number SRP030024] was sequenced [Roche 454 GS FLX Ti sequencer (454 Life Sciences, Branford, CT, USA) at LifeSequencing S.L., Valencia, Spain], analysed and compared with the sequences in the NCBI non-redundant public database (Altschul *et al*., 1997). Thirty-one predicted open reading frames were identified (Lukashin and Borodovsky, 1998); one encoded a putative esterase (CN1E1) with the α/β hydrolase fold. The protein [311 amino acids (AA); molecular weight (MW) 33 146 Da; isoelectric point (*p*I) 6.34] was produced in a soluble form (Fig. 2) upon expression in the pET-46 Ek/LIC vector (using the forward and reverse primers 5′-GACGACGACAAGATGGCGGTAGATCCG-3′ and 5′-GAGGAGAAGCCCGGTTATCTCGGTCCGGC-3′ respectively) and *E. coli* BL21 (DE3) (conditions described in Alcaide *et al*., 2013). The sequence was analysed, which indicated that it belonged to the α/β hydrolase superfamily; the esterase/lipase-like protein from *Paenibacillus mucilaginosus* 3016 (YP_005313749.1) is the most similar enzyme (AA sequence identity: 54%; similarity: 68%). It was also homologous (≤ 56% identity) to predicted esterases/lipases from uncultured microorganisms derived from soil and marine sediment samples (Fig. 3). This enzyme can be categorized in the microbial family IV described by Arpigny and Jaeger (1999); it includes a typical -GlyxSerxGly-motif and presumptive Ser-Asp-His catalytic triad (Ser155, Asp250 and His280). CN1E1 was structurally most similar to a lipase/esterase-like protein from *Alicyclobacillus acidocaldarius* [identity: 45%; Protein Data Bank (PDB) code 2HM7_A].

**Figure 1:**
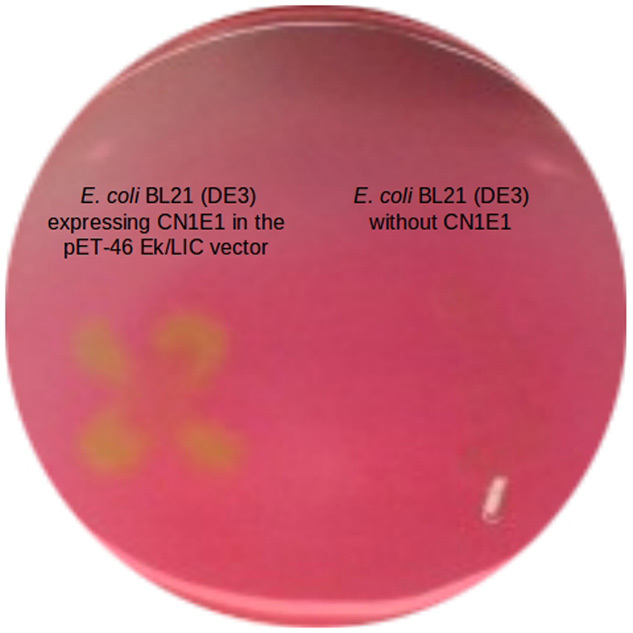
Hydrolytic phenotype for *E. coli* BL21 (DE3) that either expressed or did not express CN1E1 using the pET-46 Ek/LIC vector. The cells were plated on fresh Luria Bertani (LB) plates with ampicillin (50 μg ml^−1^). The plates [with 1.0 mM isopropyl-β-D-galactopyranoside (IPTG)] were incubated for 12 h at 37°C and then covered with a second layer that included the substrate [20 ml 5 mM *N*-(2-hydroxyethyl) piperazine-*N′*-(3-propanesulfonic acid) (EPPS) buffer, pH 8.0, 0.4% agarose and 320 μl of a naphthalene carboxylic acid methyl ester solution in acetonitrile (100 mg ml^−1^)]. The active phenotype was apparent due to the colour change resulting from substrate hydrolysis and acetic acid formation, which changes the pH indicator from red to yellow.

**Figure 2:**
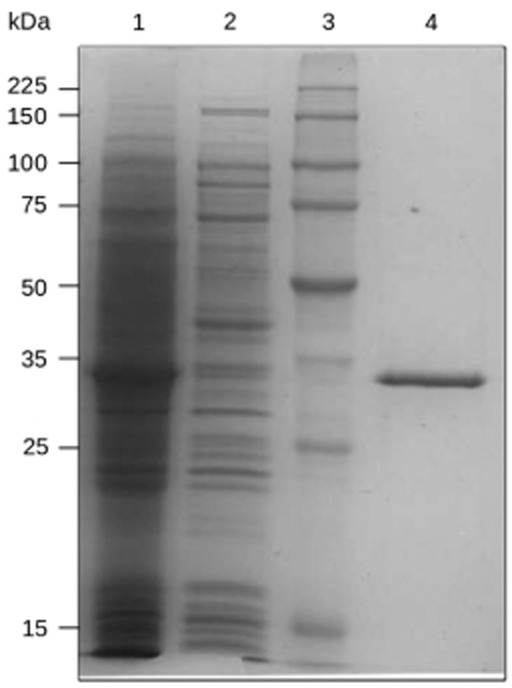
A Coomassie-stained SDS-PAGE gel showed that the active form of the protein CN1E1 was over-expressed in *E. coli* at 16°C. The expression and purification conditions were reported in Martínez-Martínez and colleagues (2013). As shown, a high percentage of the protein was produced in its soluble form, which yielded a purity greater than 98% after a single His_6_-tag purification step. Abbreviation: MW, molecular weight marker. The lanes include the following: lane 1, soluble cell fraction after induction with 1.0 mM IPTG; lane 2, soluble cell fraction without IPTG; lane 3, molecular weight marker (from top to bottom: 225, 150, 100, 75, 50, 35, 25 and 15 kDa); and lane 4, pure protein after His_6_-tag purification.

**Figure 3:**
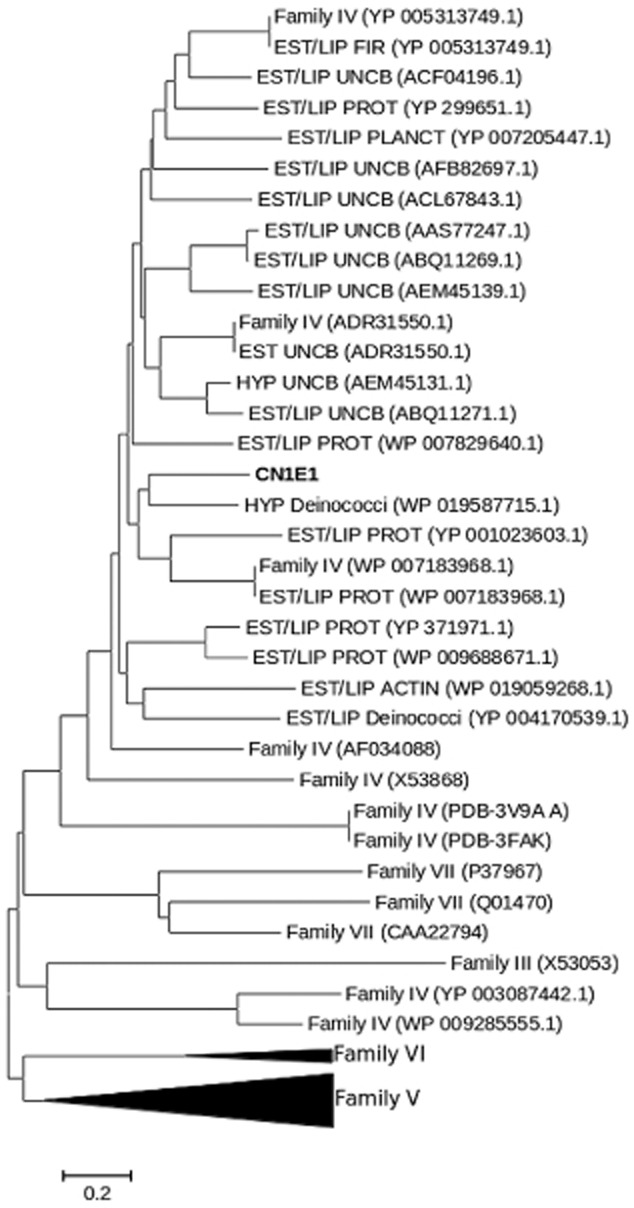
The unrooted circular neighbour-joining tree indicates the polypeptide sequence phylogenetic positions for the CN1E1 enzyme (in boldface) and reference hydrolases. The tree was constructed using an aligned 297 AA-long sequence. The GenBank and PDB accession numbers are indicated in brackets. For the dendrogram construction details, see Martínez-Martínez and colleagues (2013) and Tamura and colleagues (2007). The Family V cluster includes the sequences with the following accession numbers: YP960710.1, ADP98993.1, ZP01735705.1, ZP01735705.1, CAE54381.1, ZP01307774.1, X53869, AEO74498.1, YP001347584.1, NP251639.1, YP790224.1, YP0029804424.1, YP002909304.2, YP001810250.1, ZP03570306.1, YP299126.1, YP583166.1, YP002005156.1, 725653.1 and YP006884923.1. The Family VI cluster includes YP007541785, WP007625024, WP008294645, S78600 and PDB 3CN7. The scale bar represents 0.2 substitutions per position. The lipase/esterase families are depicted based on the Arpigny and Jaeger (1999) classification system. The abbreviations are as follows: EST, esterase; LIP, lipase; HYP, hypothetical protein; ACTIN, Actinobacteria; FIR, Firmicutes; PLANCT, Planctomycetes; PROT, Proteobacteria; UNCB, uncultured bacterium.

The pure enzyme was most active at 25–30°C and pH 8.5 (Fig. 4); it retained approximately 55% of its activity at 4°C and less than 8% at ≥ 55°C, which indicates that it is a cold-adapted enzyme. The substrates used herein included 12 model esters [6 *p*-nitrophenol (*p*NP) and 6 triacylglycerol] and 86 structurally different esters (Martínez-Martínez *et al*., 2013). Based on the specific activities determined (units g^−1^; one unit is the amount of enzyme that hydrolyses 1 μmol of substrate per min under the assay conditions, as reported by Martínez-Martínez *et al*., 2013), CN1E1 showed a capacity for accepting 61 esters with different alcohol and acid moieties (Fig. 5). Considering the acyl chain length and *p*-nitrophenyl ester hydrolysis, CN1E1 was most active with *p*NP-acetate (∼ 55 000 units g^−1^ at 40°C and pH 8.0); it also hydrolysed *p*NP-dodecanoate, albeit at a rate of four orders of magnitude lower. As shown in Fig. 5, *p*NP esters were the preferred substrates among the examined esters. This enzyme hydrolysed short-chain triacylglycerols that ranged from triacetin (which was preferred; ∼ 23 000 units g^−1^ at 40°C and pH 8.0) to tricaproin (∼ 21 units g^−1^) and short-chain halogenated and non-halogenated alkyl and aryl esters; methyl bromoacetate (∼ 31 000 units g^−1^) and butyl acetate (∼ 11 700 units g^−1^) were preferred respectively (Fig. 5). Because the enzyme preferred short-chain triacylglycerols and short-to-medium size alkyl and aryl esters, the α/β-hydrolase is likely an esterase. It also showed a capacity for accepting tri-*O*-acetyl-glucal (∼ 12 500 units g^−1^), the carbohydrate ester α-D-glucose pentaacetate (∼ 373 units g^−1^) and hydroxycinnamic acid-like esters, such as 2,5-dihydroxycinnamic acid methyl ester (∼ 10 700 units g^−1^), methyl cinnamate (∼ 28 units g^−1^) and caffeic acid phenethyl ester (∼ 19 units g^−1^) (Fig. 5). Such characteristics suggest that the enzyme can support polysaccharide degradation. Amino acid esters, such as L-proline and L-serine esters, were also hydrolysed (∼ 24–1.2 units g^−1^); however, they were among the non-preferred substrates (Fig. 5). Under our assay conditions, the esterase was also active (from ∼ 609 to 5.2 units g^−1^) and enantio-selective for (±)-menthyl-acetate, (±)-methyl lactate, (±)-neomenthyl-acetate, (±)-methyl-mandelate and (±)-glycidyl 4-nitrobenzoate with an (*S*)-preference (Fig. 5). γ-Butyrolactone (∼ 141 units g^−1^), γ-valerolactone (∼ 81 units g^−1^) and (±)-pantolactone [the (*R*) enantiomer was preferred; ∼ 606 units g^−1^] were also accepted as substrates (Fig. 5).

**Figure 4:**
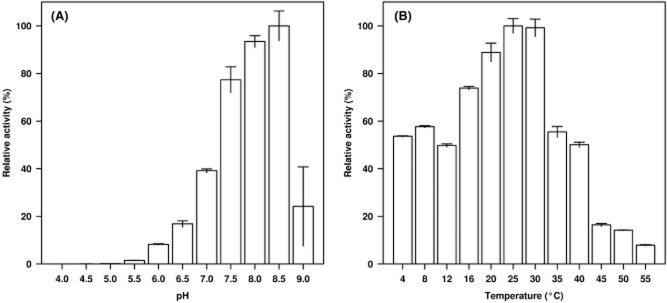
The wild-type CN1E1 α/β hydrolase pH (A) and temperature (B) profiles. Esterase activity using *p*NP-propionate (at 410 nm) was determined as described previously (Martínez-Martínez *et al*., 2013). The standard deviation (SD) for the triplicate assays is shown. We examined pH values between 4.0 and 9.5 and temperatures between 4 and 80°C to determine the optimal parameters. The following buffers were examined at 40 mM: sodium citrate (pH 4.0–4.5), sodium acetate (pH 5.0–6.0), 2-(N-morpholino)ethanesulfonic acid (MES) (pH 5.5–6.0), piperazine-N,N′-bis(ethanesulfonic acid) (PIPES) (pH 6.0–7.0), 4-(2-hydroxyethylpiperazine-1-ethanesulfonic acid (HEPES) (pH 7.0–8.0), K/Na-phosphate (pH 7.5), Tris-HCl (pH 8.5) and glycine (pH 9.0–9.5). The pH was adjusted at 25°C. The pH and temperature profiles were collected at 40°C (panel A) and pH 7.0 (using 40 mM HEPES; panel B) respectively. In panels (A) and (B), 100% of the activity refers to 27.08 ± 1.80 and 21.17 ± 1.41 units mg^−1^ respectively.

**Figure 5:**
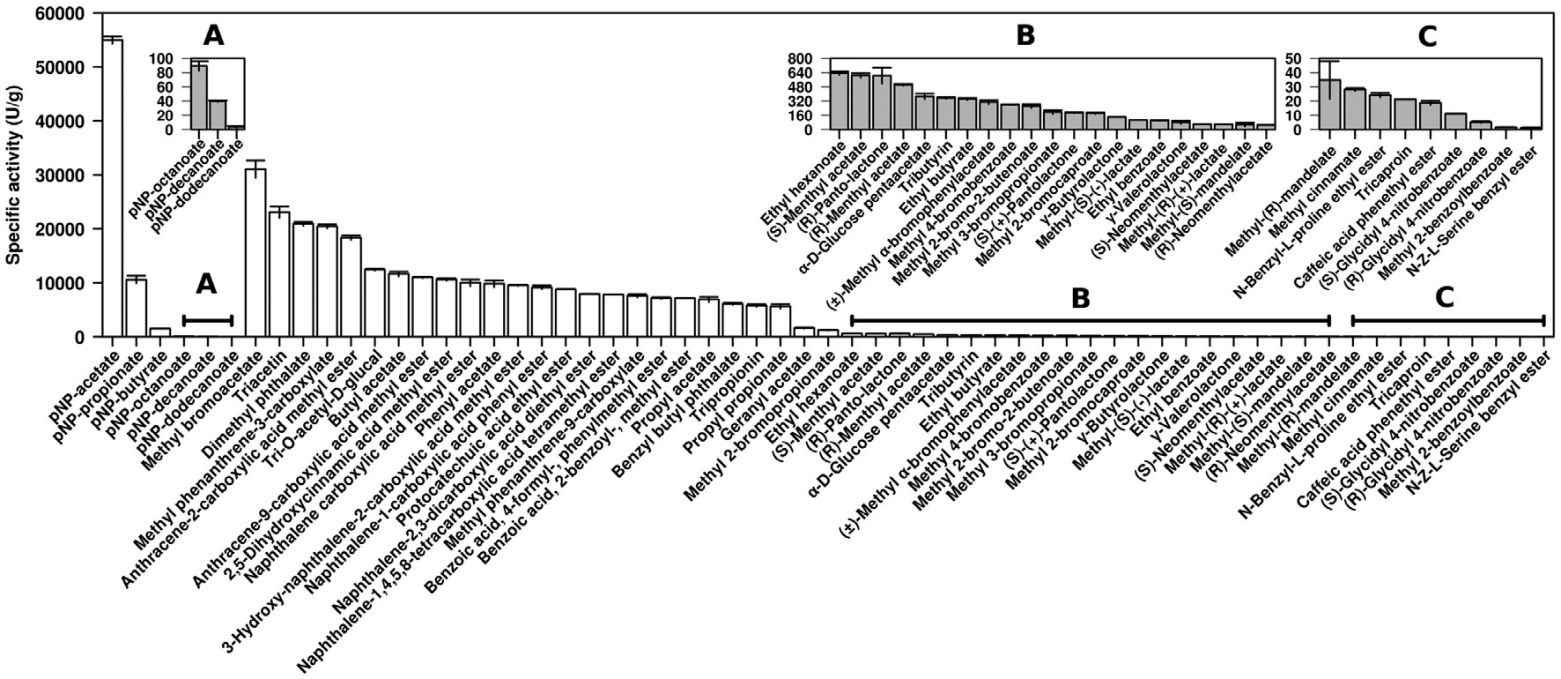
Substrate profile for the wild-type CN1E1 α/β hydrolase using a set of structurally diverse esters. The specific activities were calculated in triplicate as described by Martínez-Martínez and colleagues (2013) at 40°C in 20 mM HEPES buffer pH 7.0 (for *p*NP esters) or 5 mM EPPS buffer pH 8.0 (for the remaining esters). The standard deviation (SD) for the triplicate assays is shown. The chemicals used for the enzymatic experiments were the purest grade available and were purchased from Fluka-Aldrich-Sigma Chemical Co. (St. Louis, MO, USA). The hydroxycinnamic-like esters were supplied by Apin Chemicals (Oxon, UK), the methyl phenanthrene-3-carboxylate and methyl phenanthrene-9-carboxylate were supplied by Wuhan Farthest Chemical (Mainland, China), and the anthracene-9-carboxylic acid methyl ester and anthracene-3-carboxylic acid methyl ester were obtained from Alfa Aesar (Karlsruhe, Germany). Insets (A) to (C) in the figure represent a zoom for substrates hydrolysed at low rates.

Because the active clone with CN1E1 actively hydrolysed naphthalene carboxylic acid methyl ester on an agar plate (Fig. 1), we further tested and quantified hydrolysis for the pure enzyme using this and other model PAH esters; the pH-indicator protocol (at 40°C and pH 8.0) used to examine hydrolysis for the above esters was also used for this experiment (Martínez-Martínez *et al*., 2013). CN1E1 showed broad reactivity towards PAH-like esters, including phthalate alkyl and aryl (benzene) esters (from ∼ 21 000 to 6100 units g^−1^), alkyl esters of phenanthrene (from ∼ 20 500 to 7600 units g^−1^), anthracene (from ∼ 18 400 to 11 000 units g^−1^), naphthalene (from ∼ 10 000 to 7800 units g^−1^) and protocatechuate (∼ 8800 units g^−1^) and benzoate esters with substituent phenyl/benzene rings (from ∼ 7600 to 7200 units g^−1^). The majority of such compounds were among the best substrates for CN1E1 (Fig. 5); this enzyme showed regio-selectivity for phenanthrene-3-carboxylate over methyl phenanthrene-9-carboxylate (2.7-fold) and, to a lesser extent, for anthracene-2-carboxylate over anthracene-9-carboxylate (1.7-fold). Although previous reports have shown that other esterases can degrade phthalate esters (Maruyama *et al*., 2005; Saito *et al*., 2010; Wu *et al*., 2013), to the best of our knowledge, the specialized literature has not reported an esterase with the capacity to degrade PAH esters with multiple aromatic (e.g. benzene) rings.

The half-saturation (Michaelis) coefficient (*K*_m_), catalytic rate constant (*k*_cat_) and catalytic efficiency (*k*_cat_/*K*_m_) values were determined for the 14 best and structurally distinct substrates (Table 1). The parameters were determined as described previously (Alcaide *et al*., 2013) at pH 8.0 and 40°C (except for *p*NP-acetate were pH 7.0 was used to ensure substrate stability). The substrates analysed include *p*NP-acetate (the model p-nitrophenyl ester), triacetin (the model triacylglycerol), methyl bromoacetate (the model halogenated ester), tri-*O*-acetyl-glucal (the model polysaccharide-like ester), butyl acetate (the model alkyl ester), phenyl acetate (the model aryl ester), 2,5-dihydroxycinnamic acid methyl ester (the model hydroxycinnamic-like ester) and seven chemically distinct model PAH esters. As shown in Table 1, for catalytic efficiencies, *p*NP-acetate and triacetin were the most and least efficient substrates {[(*k*_cat_/*K*_m_)]*_p_*_NPacetate_/[(*k*_cat_/*K*_m_)]_triacetin_ ∼36/1 factor}. The catalytic efficiency (*k*_cat_/*K*_m_) for PAH-like esters ranged from 4.5-(for methyl phenanthrene-9-carboxylate) to 13.3-fold (for dimethyl phthalate) lower than for *p*NP-acetate, primarily due to a significant decrease in *K*_m_ values (from 2.8-to 11.1-fold, depending on the substrate) for the *p*NP-acetate. The *k*_cat_/*K*_m_ values were used to establish the following order: phenanthrene > naphthalene > benzoic > protocatechuic > anthracene > phthalate. In addition, the enzyme's marked regio-selectivity was demonstrated by a ∼ 3.0-fold higher efficiency for methyl phenanthrene-9-carboxylate compared with the 3-carboxylate ester.

**Table 1:** The half-saturation (Michaelis) coefficient (*K*_m_), catalytic rate constant (*k*_cat_) and catalytic efficiency (*k*_cat_/*K*_m_) values for the purified CN1E1 esterase for representative, structurally diverse esters. Three independent experiments at 40°C and pH 8.0 (5 mM EPPS buffer) were performed for each parameter, and the data are shown with the standard deviation. The parameters were determined using conditions and methods described previously (Alcaide *et al*., 2013)

Substrate	*K*_m_ (mM)	*k*_cat_ (s-^−1^)	*k*_cat_/*K*_m_ (s^−1 ^M^−1^)
*p*NP-acetate	0.19 ± 0.01	7.52 ± 0.21	39 867
Methyl phenanthrene-9-carboxylate	0.53 ± 0.03	4.76 ± 0.29	8 947
Naphthalene carboxylic acid methyl ester	0.71 ± 0.06	5.82 ± 0.24	8 156
Tri-*O*-acetyl-D-glucal	1.08 ± 0.05	7.81 ± 0.47	7 237
Phenyl acetate	0.94 ± 0.05	5.75 ± 0.40	6 096
Benzoic acid, 4-formyl-, phenylmethyl ester	0.64 ± 0.06	3.61 ± 0.17	5 626
Protocatechuic acid ethyl ester	1.14 ± 0.06	5.49 ± 0.33	4 798
2,5-Dihydroxycinnamic acid methyl ester	1.06 ± 0.06	5.07 ± 0.17	4 790
Butyl acetate	2.10 ± 0.17	8.76 ± 0.48	4 167
Anthracene-2-carboxylic acid methyl ester	1.47 ± 0.05	5.59 ± 0.20	3 802
Methyl phenanthrene-3-carboxylate	1.60 ± 0.09	4.85 ± 0.18	3 022
Dimethyl phthalate	1.59 ± 0.10	4.75 ± 0.17	2 998
Methyl bromoacetate	1.70 ± 0.09	4.01 ± 0.14	2 358
Triacetin	1.64 ± 0.08	1.82 ± 0.10	1 109

Taken together, the results indicate that the esterase CN1E1 from a PAH-degrading microbial consortium includes a broad and unusual substrate profile. Three-dimensional modelling (not shown) predicts a broad molecular environment in the CN1E1 active site, which is consistent with high-substrate accessibility and the specific activity and catalytic efficiency values for multiple multi-aromatic substrates (Fig. 5 and Table 1). The observed functions may indicate metabolic and ecological capacities *in vivo* and provide research avenues for biotechnology applications. From an ecological perspective, the protein, which is likely an esterase/lipase, hydrolysed not only common esters but also (±)-pantolactone, γ-butyrolactone and γ-valerolactone; thus, CN1E1 may also be a lactone hydrolase. γ-Valerolactone hydrolases play a key role in degrading cyclopentanol (Iwaki *et al*., 2002), and the organism that produces CN1E1 may use cyclopentanol or cyclopentane as carbon sources. Notably, this protein also hydrolysed benzoic acid, 2-benzoyl-and methyl ester (Fig. 5), which has been proposed as an intermediate in the phenanthrene degradation pathway (Luan *et al*., 2006). In addition, this enzyme showed a high activity for both alkyl and benzyl groups on phthalate esters, which indicates that the esterase may also be involved in hydrolysing phthalate esters. Although ester formation is a common detoxification mechanism (e.g. Luan *et al*., 2006), little is known about whether PAH esters occur naturally. Because most of the aromatic esters investigated herein are known polymer components for plastic and pesticides, the results indicate that organism with the CN1E1 enzyme may initiate degradation of such recalcitrant molecules, which may be introduced into ecosystems through anthropogenic activities or produced at intermediate stages in biodegradation pathways (Luan *et al*., 2006). A compositional similarity analysis using GOHTAM (Ménigaud *et al*., 2012) suggests that such organism is a α-proteobacterium in the order Rhizobiales (most likely *Mesorizhobium*); this was the only Rhizobiales member in the CN1 community detected by both full-length and partial 16S rRNA gene sequences analysis (Guazzaroni *et al*., 2013). Herein, we have enhanced our understanding of soil bacterial systems by demonstrating that aromatic molecule transformation mediated by esterases/lipase-like proteins may facilitate a deep breakdown of PAH components.

Our data show that CN1E1 is, to the best of our knowledge, the first efficient and catalytically active esterase from the α/β-hydrolase family for PAH ester hydrolysis; this suggests that this protein may be applied to generate a unique set of complex *a la carte* aromatic molecules with improved or unknown properties (Storms and Farrar, 1995; Noh *et al*., 2001; Chae *et al*., 2002; Yen *et al*., 2005; Jones and Sumner, 2006; Zhu *et al*., 2011; Kita *et al*., 2012; Maruyama *et al*., 2012). We believe that CN1E1 may expand the enzyme toolbox for new biotechnological opportunities involving heterocyclic aromatic compounds for future studies due to the inherent properties of enzymes compared with chemical heterogeneous-based processes and the unusual substrate range and preference and regio-and enantio-selective properties of CN1E1 compared with previously reported esterases.
